# Nutraceutical Profiling, Bioactive Composition, and Biological Applications of *Lepidium sativum* L.

**DOI:** 10.1155/2022/2910411

**Published:** 2022-01-19

**Authors:** Sakshi Painuli, Cristina Quispe, Jesús Herrera-Bravo, Prabhakar Semwal, Miquel Martorell, Zainab M. Almarhoon, Ainur Seilkhan, Alibek Ydyrys, Javad Sharifi Rad, Mohammed M. Alshehri, Sevgi Durna Daştan, Yasaman Taheri, Daniela Calina, William C. Cho

**Affiliations:** ^1^Himalayan Environmental Studies and Conservation Organization, Dehradun, 248006 Uttarakhand, India; ^2^Facultad de Ciencias de la Salud, Universidad Arturo Prat, Avda. Arturo Prat 2120, Iquique 1110939, Chile; ^3^Departamento de Ciencias Básicas, Facultad de Ciencias, Universidad Santo Tomas, Chile; ^4^Center of Molecular Biology and Pharmacogenetics, Scientific and Technological Bioresource Nucleus, Universidad de La Frontera, Temuco 4811230, Chile; ^5^Department of Life Sciences, Graphic Era Deemed to Be University, Dehradun, 248 002 Uttarakhand, India; ^6^Department of Nutrition and Dietetics, Faculty of Pharmacy and Centre for Healthy Living, University of Concepción, 4070386 Concepción, Chile; ^7^Department of Chemistry, College of Science, King Saud University, P. O. Box 2455, Riyadh 11451, Saudi Arabia; ^8^Educational Program, Geography, Environment and Service Sector, Abai Kazakh National Pedagogical University, Almaty, Kazakhstan; ^9^Biomedical Research Centre, Al-Farabi Kazakh National University, Almaty, Kazakhstan; ^10^Phytochemistry Research Center, Shahid Beheshti University of Medical Sciences, Tehran, Iran; ^11^Pharmaceutical Care Department, Ministry of National Guard-Health Affairs, Riyadh, Saudi Arabia; ^12^Department of Biology, Faculty of Science, Sivas Cumhuriyet University, 58140 Sivas, Turkey; ^13^Beekeeping Development Application and Research Center, Sivas Cumhuriyet University, 58140 Sivas, Turkey; ^14^Department of Clinical Pharmacy, University of Medicine and Pharmacy of Craiova, 200349 Craiova, Romania; ^15^Department of Clinical Oncology, Queen Elizabeth Hospital, Kowloon, Hong Kong

## Abstract

The roots, leaves, and seeds of *Lepidium sativum* L., popularly known as Garden cress in different regions, have high economic importance; although, the crop is particularly cultivated for the seeds. In traditional medicine, this plant has been reported to possess various biological activities. This review is aimed at providing updated and critical scientific information about the traditional, nutritional, phytochemical, and biological activities of *L. sativum*. In addition, the geographic distribution is also reviewed. The comprehensive literature search was carried out with the help of different search engines PubMed, Web of Science, and Science Direct. This review highlighted the importance of *L. sativum* as an edible herb that possesses a wide range of therapeutic properties along with high nutritional values. Preclinical studies (in vitro and in vivo) displayed anticancer, hepatoprotective, antidiabetic, hypoglycemic, antioxidant, antimicrobial, gastrointestinal, and fracture/bone healing activities of *L. sativum* and support the clinical importance of plant-derived bioactive compounds for the treatment of different diseases. Screening of literature revealed that *L. sativum* species and their bioactive compounds may be a significant source for new drug compounds and also could be used against malnutrition. Further clinical trials are needed to effectively assess the actual potential of the species and its bioactive compounds.

## 1. Introduction

A large number of people or community from developed and developing countries depend on medicinal plants for treatments, skin care, cultural progress, and economic growth [1, 2]. The World Health Organization (WHO) projected that 80% of the world's population relies on traditional medicines, and around 19.4 billion global revenue were recorded for herbal remedies in 2010 [3, 4]. The market demand for medicinal plants is increasing continuously and according to WHO the demand will be more than the US $ 5 trillion in 2050 [5].

*Lepidium sativum* L. popularly known as garden cress in different regions of the world is an edible annual and fast-growing herb belongs to the family Brassicaceae and genus *Lepidium* [6]. The genus consists of more than 175 species around the world; among them, several species are known for their nutritional and therapeutic properties [7, 8].

From prehistoric time, *L. sativum* has been consumed by ancient Egyptians and Romans for several health-promoting benefits [9]. Traditionally, *L. sativum* is used for the treatment of various diseases like asthma, tumors of the uterus, ulcers, hemorrhoidal haemorrhage, coughing, wounds, dermatomycosis, dysmenorrhea, sciatica, and nasal polyps. The seeds of this species have been utilized as a galactagogues and abortive agent and are also used to treat sore throat, headache, cough, asthma, malaria, syphilis, and impotence [10]. A seed paste prepared in water is used for skin problems and sunburns while the mucus of seeds is used against diarrhoea and irritation of the intestines in dysentery, and germinating seeds are used for constipation [11, 12]. The leaves of *L. sativum* are diuretic, mildly stimulant, and also used in liver problems and scorbutic diseases [13].

People consume it in the form of salad, sprouts, and spicy seasoning, and the oil extracted from their seed is used for seasoning [14, 15]. Different parts such as roots, leaves, and seeds of this plant species have immense economic importance; although, the crop is particularly cultivated for the seeds [16].

*L. sativum* has been reported to possess various biological activities such as antimicrobial, bronchodilator, hypotensive, allopathic, hypoglycemic, hepatoprotective, antioxidant, and against hiccup [17–21]. Its mucilage possesses various characteristics such as gelling, binding, and disintegrating, which help in the development of desirable pharmaceutical dosage forms [16]. The phytochemical profiling of *L. sativum* showed the presence of flavonoids, phenols, cardiotonic glycosides, cardiac glycosides, alkaloids, coumarins, proteins, and amino acids [10].

The purpose of the present review is to provide updated and quantified scientific information about the traditional, nutritional, phytochemical, and biological activities of *L. sativum*.

## 2. Methodology

For this review, we collected literature published in English from scientific databases such as PubMed, Web of Science, and ScienceDirect, before July 2021 on phytochemistry, nutritional profile, and pharmacology of Lepidium sativum species. The following MESH terms were used for searching: “Lepidium sativum/chemistry,” Seeds/chemistry, Oxidative Stress/drug effects, “Plant Extracts/chemistry,” “Plant Extracts/pharmacology,” “Antineoplastic Agents,” “Antioxidants/chemistry,” “Antioxidants/pharmacology,” “Apoptosis/drug effects,” “Cell Line, Tumor,” “DNA Damage/drug effects,” “Flavonoids/chemistry,” “Flavonoids/pharmacology,” “Blood Glucose,” “Diabetes Mellitus,” “Experimental/drug therapy,” “Hyperglycemia/drug therapy,” “Hypoglycemic Agents/pharmacology,” “Animals,” and “Humans.”

All selected papers were analyzed and summarized to prepare this comprehensive review.

The plant taxonomy was verified by the database “The PlantList,” and the chemical formulas were validated with Chemspider [22, 23].

## 3. Bioactive Compounds

Active compounds or secondary metabolites are produced in plants as a byproduct of various metabolic reactions; although they do not play a primary role in plant reactions, they are important in many plant defence mechanisms and are also known for their biological or therapeutic activities [24–26]. The most important class of secondary metabolites are phenols, flavonoids, terpenoids, alkaloids, saponins, and glycosides [27–29].

Phytochemically, the seeds, leaves, roots, and seed oil of *L. sativum* are a rich source of alkaloids, glucosinolates, saponins, terpenes, saturated, and essential fatty acids [13, 30–33].

Glucosinolates are a wide group of secondary metabolites consisting of sulphur and nitrogen molecules and are mainly known for their nutritional effects and other therapeutic properties like antimicrobial, antioxidant, anticancer, and anti-inflammatory [34, 35].

Total phenolic and flavonoid content of *L. sativum* leaves of two cultivars (Dadas and Izmir from Turkey) was measured to be 0.573 mg gallic acid equivalent (GAE)/g fresh weight (FW) and 6.332 mg GAE/g DW for Dadas cultivar and 0.774 mg GAE/g FW and 7.401 mg GAE/g DW for Izmir cultivar, respectively [36]. The ascorbic acid content for *L. sativum* leaves was measured to be 54 mg/100 g FW and 74 mg/100 g FW for Dadas and Izmir cultivars [36]. However, the methanolic extract of seeds showed the presence of 0.5% and 0.375% of phenolic and flavonoid content [32].

Malar et al. [37] reported the ascorbic acid content in stem (11.74 ± 0.83 mg), leaves (7.4 ± 0.38 mg), whole plant (12.5 ± 0.60 mg), and seeds (9.68 ± 0.72 mg) of *L.* sativum.

Chatoui et al. [38] showed the presence of tannin in the ethanolic and methanolic seed extract of *L. sativum* collected from different regions of Morocco. The maximum tannin acid (31.50 ± 0.11 mg catechin/g extract) was observed in methanolic seed extract of *L. sativum* of Ben-Ahmed region, Morocco, whereas the minimum (8.33 ± 0.11 mg catechin/g extract) amount of tannin was measured in the ethanolic extract of *L. sativum* of Rommani region, Morocco [38]. Other studies from different regions also showed that *L. sativum* has a significant amount of phenolic and flavonoid content (Table 1).

Regarding the essential oil composition, Afsharypuor and Hadi [45] identified the presence of 1,8-cineole, benzyl isothiocyanate, *α*-pinene, and phenyl acetonitrile in seeds, benzyl isothiocyanate, *α*-pinene, palmitic acid, and linoleic acid in roots, and benzyl isothiocyanate, *α*-pinene, palmitic acid, phenyl acetonitrile, sabinene, and limonene, *β*-thujone in the aerial part of *L. sativum* by gas chromatography-mass spectrometry (GC-MS) analysis [45].

The seeds of *L. sativum* are comprised of 24% oil which contains linoleic acid and *α*-linoleic acid. It is reactively more stable due to the presence of phytosterols and antioxidant content [46, 47].

**Figure 1:**
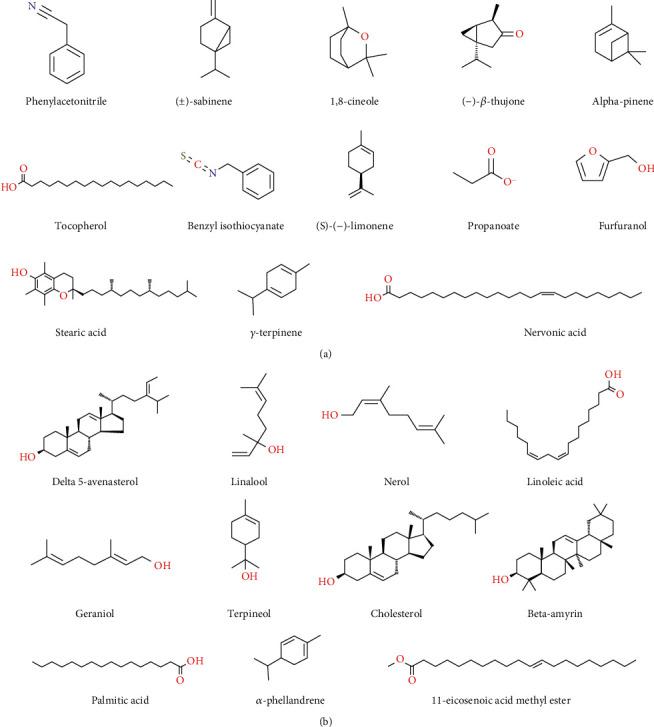
(a) Chemical structure of several bioactive compounds present in essential oil of *L. sativum.* (b) Chemical structure of several bioactive compounds present in the essential oil of *L. sativum*.

Singh et al. [48] reported the presence of 2-pentanoic acid, penta-decadienoic acid, pentanoic acid, succinic acid, butyric acid, acetic acid, oxalic acid, carbonic acid, propanoic acid, and cyclohexane carboxylic acid in the seed oil of *L. sativum*. The chemical structures of bioactive compounds present in the essential oil of the species are shown in Figures 1(a) and 1(b) while a detail description of essential oil composition has been presented in Table 2.

The analyses of the chemical composition of *L. sativum* extract revealed the presence of five glucosinolates in seeds (glucotropaeolin and 2-phenyl ethyl glucosinolate) and fresh herb (glucotropaeolin, methyl glucosinolate, 2-ethyl butyl glucosinolate, and butyl glucosinolate) [56]. Williams et al. (2009) reported the presence of glucotropaeolin as a principal glucosinolate and gluconasturtiin in the seeds of *L. sativum* [57]. Hussain et al. (2011) [58] reported the presence of 19 phytochemicals in the methanolic leaves to extract *L. sativum* including campesterol, cis-vaccenic acid, 2-naphthalenol, 1-nitro-2-propanol,1-deoxy-d-mannitol, allyl isothiocyanate, and paromomycin, among others.

Maier et al. [59] identified the imidazole alkaloid lepidine along with five new dimeric (lepidines B, C, D, E, and F) and two monomeric (semilepidinosides A and B) imidazole alkaloids in seeds of *L. sativum* [59], while the presence of 10 major compounds includes benzyl nitrile, 2,3,4-tri-methoxycinna-mic acid, 5-hydroxy-methyl furfural, and furfural was reported by El-Gendy [60].

A complete screening of phytochemicals present in *L. sativum* seeds was evaluated by ultrahigh-performance liquid chromatography (UHPLC)/photodiode array detection (PDA)/electrospray ionization-mass spectroscopy (ESI-MS) method as well as head space solid-phase microextraction (SPME)-GC/MS methods [61]. A total of 32 metabolites from flavonoid, glucosinolate, phenolic acid, sugar, coumarin, lignan, glycoalkaloid, steroid, and fatty acid classes were identified via UHPLC/PDA/ESI-MS, and 66 metabolites from alcohol, acid, ester, aromatic, ketone, aldehyde, monoterpene hydrocarbon, and among other classes were identified by (SPME)-GC/MS [61]. All the above studies are reported from different regions including Saudi Arabia, India, Egypt, and Iraq, which indicates that the leaves, seed, or seed oil of *L. sativum* could be a valuable source of important active compounds with significant biological activity.

**Figure 2:**
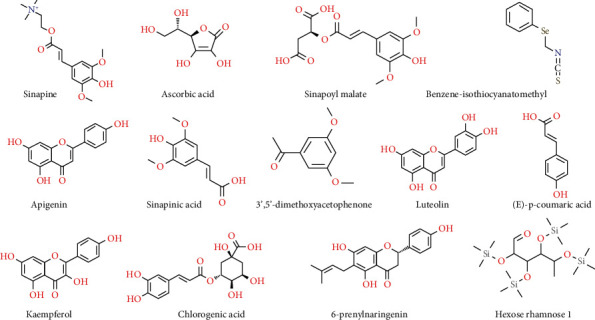
Chemical structure of bioactive compounds present in *L. sativum* extracts.

The chemical structure of bioactive compounds present in the extracts of *L. sativum* has been displayed in Figure 2 while a detailed description of bioactive compounds present in different parts of the species has been presented in Table 3.

## 4. Nutritional Profile

*L. sativum* is considered a valuable source of nutrition with significant therapeutic properties. In the last few years, several researchers from different regions have investigated the nutritional profiling of the leaves, seed, and seed oil of *L. sativum* (Tables 4 and 5).

Hassan et al. [68] evaluated that in *L. sativum* leaves, the highest amount of mineral value was observed for potassium (1850.00 ± 43.30 mg/100 g dry weight (DW)) followed by calcium (829.13 ± 20.70 mg/100 g DW), and the minimum value was observed for chromium (0.36 ± 0.27 mg/100 g DW); however, the maximum amino acid content in leaves was calculated for glutamic acid (9.36 ± 0.06 g/100 g protein DW), and minimum value was shown by cysteine (0.42 ± 0.20 g/100 g protein DW) [58].

In three studies from different regions (Nigeria, Saudi Arabia, and Pakistan), the highest mineral value of *L. sativum* seed was calculated for potassium (1193.95 ± 10.51; 785.0 ± 7.51; 1236.5 ± 1.67 mg/100 g) followed by phosphorus (514.59 ± 10.67; 616.50 ± 9.67; 608.63 ± 1.39 mg/100 g) [41, 69, 70], and the minimum mineral value was observed for molybdenum (0.43 ± 0.08 mg/100 g) [70].

The amino acid analyses showed different results in terms of the maximum and minimum amino acid value, and it was recorded for glutamic acid (19.33 ± 0.19 g/100 g protein) and methionine (0.97 ± 0.02 g/100 g protein) [70]; however, in another study, the highest amino acid value was measured for leucine (9.03 ± 0.007 g/100 g protein), and lowest amino acid values were measured for cysteine (0.80 ± 0.00 g/100 g protein) [69].

The estimation of fatty acid was done for three seed oil extracts of *L. sativum* prepared from the cold press extraction method, Soxhlet extraction method, and supercritical carbon dioxide extraction method. The study findings showed that in all the seed oil extracts, the maximum fatty acid content was measured for linoleic acid (~34-35%), and the minimum was observed in oleic acid (~2.8%) [46].

**Figure 3:**
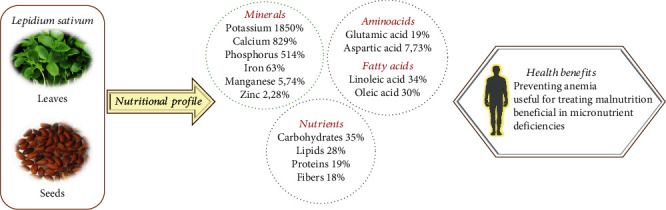
The most representative nutritional compounds of *Lepidum sativum* and the correlation with their beneficial effects for human health.

The nutritional profiling showed that the leaves, seeds, and seed oil of *L. sativum* possess appropriate nutritional content which can help in combating anemia, malnutrition, and several micronutrient deficiencies (Figure 3).

## 5. Pharmacological Properties

The major role of food is to fulfil the requirement of necessary nutrients in the body and to satisfy hunger; however, nowadays, food from edible plants also plays a significant role in preventing and curing several diseases and disorders due to the presence of different bioactive compounds [75]. The species comprise a variety of bioactive compounds along with strong nutraceutical potential and showed several biological activities [76]. In this section, we discussed different biological applications of the species including anticancer, hepatoprotective, antidiabetic and hypoglycemic, antioxidant, antimicrobial, gastrointestinal, and fracture/bone healing activities.

**Figure 4:**
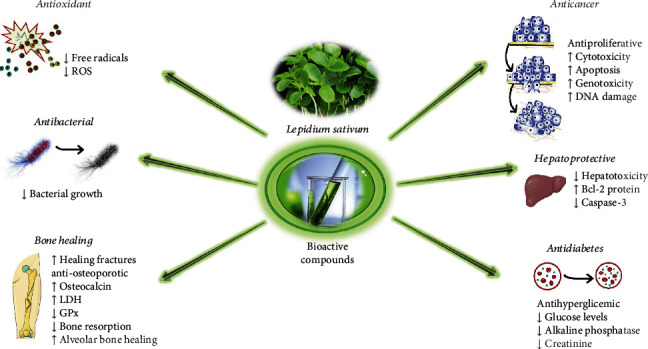
Summarized diagram with pharmacological properties of *Lepidium sativum* and its potential mechanism of actions. Abbreviations and symbols: ↑: increase; ↓: decrease; Bcl-2: B-cell lymphoma 2; GPx: glutathione peroxidase; LDH: lactate dehydrogenase; ROS: reactive oxygen species.

The most relevant pharmacological properties and their mechanisms of action are summarized in Figure 4.

### 5.1. Anticancer

Globally, cancer is the second leading cause of death and modern drugs and techniques used to treat cancer possess several toxicities and side effects [77–79]. Easily available traditional medicines and natural remedies for cancer have less or no side effects relative to modern drugs [80, 81]. Many plant extracts and plant-derived secondary metabolites are presently used to treat cancer and to eliminate the side effects of chemotherapy [82].

In the anticancer activity of *L. sativum* leave extract (aqueous) against CAL-27, a human tongue squamous carcinoma was evaluated a dose-dependent manner (70, 100, and 150 *μ*g/mL). The best result was shown at 100 and 150 *μ*g/mL of concentrations where the aqueous leaves extract of *L. sativum* caused significant damage to DNA and increase the apoptosis up to 30% and 60%. The results also showed the increase in reactive oxygen species (ROS) level in the mitochondria of CAL-27 [83]. The hydroalcoholic leave extract of *L. sativum* showed optimum antiproliferative and apoptotic activity against cervical cancer cell lines (HeLa) cell lines at 100 *μ*g/mL [84].

The combination of shoots stems and leave hydroalcoholic extracts before and after flowering was tested for cytotoxic effect against leukemia cell line (K562) at different concentrations ranging between 12.5 and 100 *μ*g/mL [85]. The hydroalcoholic extracts before and after flowering exhibit cytotoxic effect against K562 cell lines and the best results are shown at 25 *μ*g/mL of concentration.

The methanolic extract of *L. sativum* shows cytotoxic effect against lymphocyte cells and colon and endometrium cancer cell lines (DLD-1 and ECC-1) through 3-(4,5-dimethylthiazol-2-yl)-2,5-diphenyltetrazolium bromide (MTT) assay [44]. The necrotic effect, apoptotic activity, and genotoxic activity of plant extract were also investigated by lactate dehydrogenase (LDH), DNA ladder fragmentation, enzyme-linked immunosorbent assay (ELISA), ethidium bromide staining, and comet assay. The extract showed cytotoxic activity in a concentration-dependent manner against colon and endometrium cancer cells; however, the maximum apoptotic and genotoxic activity was seen at 200 *μ*g/mL of a concentration [44].

Kassie et al. [86] reported the chemoprotective effect of *L. sativum* seed extract and their compounds (glucotropaeolin and benzylisothiocyanate) on 2-amino-3-methyl-imidazo [4,5-f] quinoline- (IQ-) induced genotoxic effects and colonic preneoplastic lesions in male Fischer 344 rats. The pretreatment of the experimental model with *L. sativum* extracts (0.8 mL) and their compounds (GT: 150 mg/kg, BITC: 70 mg/kg) up to three days (consecutive) triggered a significant reduction in IQ-induced DNA damage in colon and liver cells ranging between 75 and 92% [86]. The aqueous seed extract of *L. sativum* showed cytotoxic effect against breast cancer cell lines (MCF-7) by sulforhodamine B and tryphan blue dye exclusion assay at concentration and time-dependent manner (25%, 50%, and 75%) [87].

The cytotoxic activity of seed extracts (chloroform, n-hexane, methanol, and ethyl acetate) of *L. sativum* was tested against human neuroblastoma (IMR-32), colon cancer (HT-15 and 29), and lung cancer (A-549) cell lines [88]. The study showed that all the extracts exhibited significant in vitro cytotoxicity against all the cell lines; however, methanolic seed extract shows the highest per cent of growth inhibition activity (90 ± 0.88, 95 ± 0.24, 91 ± 0.20, and 87 ± 0.65) for all the cell lines (IMR-32, HT-15, HT-29, and A-549) [88].

The aqueous seed extract of *L. sativum* with the lowest and highest concentration of 200 and 400 mg/kg was evaluated for anticancer activity against dextran sulfate sodium/azoxymethane-induced colon cancer in the albino mice model [89]. The result showed that at 400 mg/kg of concentration, the seed extract exhibits higher apoptosis and higher anticancer activity against colon cancer with a decrease in colon tumor/polyp size and incidence and tissue disorder [89]. The seed extract of *L. sativum* alone and with silver nanoparticles showed significant anticancer activity against HT-29 colon cancer cell lines by inducing apoptosis and mitotic cell arrest. They also increase the p53 expression and prevent cell division of HT-29 colon cancer cells [90].

Ait-Yahia et al. [91] studied the cytotoxic effect of aglycones (flavones/flavonoids), *C*-glycosides, and *O*-glycosides, isolated from the seed and leave extract of *L. sativum* against human laryngeal carcinoma cells (HEp2). The findings showed that all the compounds possess cytotoxic activity, whereas the highest cytotoxic effect was observed for the *O*-glycosylate rich acetate ethyl extract at 57 *μ*g/mL of concentration [91].

### 5.2. Hepatoprotective

The liver is a crucial part of the body that play a fundamental role in different physiological processes and functions including secretion, metabolism, and storage [92]. Numerous studies proved its important role in the detoxification and excretion of endogenous waste metabolites and exogenous toxic compounds from the body [93, 94].

The liver is also involved in various biochemical processes of nutrient and energy supply, growth, etc. Additionally, it helps in carbohydrate and fat metabolism, bile secretion, and vitamin storage [95, 96]. However, biological factors, genetic factors, environmental factors, autoimmune diseases, toxic compounds, and chemicals result in damage of the cell, structure, tissues, and functioning of the liver and cause hepatic diseases. Modern drugs can also cause an adverse effect on liver as they possess numerous side effects [97]. Thus, there is a need to identify the alternative treatment of hepatic diseases to discover more effective and less toxic natural agents [98–100].

Hepatoprotective activity of the seed and herb extracts (petroleum ether and alcohol) of *L. sativum* was evaluated against carbon tetrachloride- (CCl_4_-) induced toxicity in hepatocytes at different concentrations, and the results showed that both the extracts of seed and herb at a minimum concentration of 50 *μ*g/mL possess a hepatoprotective effect on the hepatocytes against CCl_4_ cytotoxicity; however, the concentration that prevents the growth of half of the cells was 150 *μ*g/mL and 200 *μ*g/mL, respectively [56]. The results also showed that the alcoholic extract is safer than petroleum ether extract [56].

*L. sativum* seed show in vivo hepatoprotective activity for the prevention of CCl_4_-induced liver damage in Wistar albino rats at different concentrations ranging from 100 mg/kg to 400 mg/kg body weight [53, 101, 102]. The total alkaloid fraction of seeds of *L. sativum* was screened for the hepatoprotective activity against CCl_4_ at 50, 150, and 250 mg/kg (i.p.) of concentrations, and the finding showed that in all concentrations, the extract showed hepatoprotective activity, and the maximum activity was observed at 250 mg/kg [103].

Sakran et al. [104] reported in vivo hepatoprotective activity of a new isoflavonoid (5,6-dimethoxy-2′,3′-methylenedioxy-7-*C*-*β*-D-gluco-pyranosyl isoflavone) isolated from the seeds of *L. sativum* against paracetamol-induced hepatotoxicity in Sprague Dawley male rats at 100 mg/kg of dose. Al-Sheddi et al. [105] reported the hepatoprotective effect of chloroform extract of seed of *L. sativum* at 5, 10, and 25 mg/mL of concentrations against hepatotoxicity induced by hydrogen peroxide in HepG2 cell lines [105].

Hepatoprotective activity of *L. sativum* seed extract (ethanolic) was evaluated at 150 and 300 mg/kg of doses against D-galactosamine/lipopolysaccharide-induced hepatotoxicity in the Wistar rat model. The result revealed the hepatoprotective activity of the *L. sativum* seed ethanolic extract and showed that the pretreatment of the extract upregulates Bcl-2 protein expression and downregulated caspase-3 in mice [21].

### 5.3. Antidiabetic

In the last few decades, the global prevalence of diabetes has risen faster not in developed but also in developing countries. Diabetes also causes dysfunction, damage, and failure of a various organ systems which can lead to premature death. Existing synthetic antidiabetic drugs show several limitations and therefore, the search for new antidiabetic agents from natural resources continues [106].

The hypoglycemic activity of aqueous seed extract of *L. sativum* was evaluated in vivo in streptozotocin-induced diabetic Wistar rats at 20 mg/kg of concentration [19, 107]. The result showed significant hypoglycemic activity in the rat model without showing any effect in basal plasma insulin concentration [19, 107].

Mishra et al. [108] also investigated the hypoglycemic activity of seeds of *L. sativum* on streptozotocin-induced diabetic Wistar rat and showed the reduction in glucose, alkaline phosphate, and creatinine levels at 20 mg/kg of dose [108]. The total alkaloid fraction of *L. sativum* seed was investigated for antidiabetic activity in alloxan-induced diabetic Wistar rat model at different (50, 150, and 250 mg/kg, i.p.) concentrations [109].

Kamani et al. [110] reported that the methanolic seed extract of *L. sativum* at 200 and 400 mg/kg of doses showed antidiabetic activity against streptozotocin-induced diabetic in albino rats. The fraction suppresses blood glucose, cholesterol, triglyceride, and urea level and showed the best antidiabetic results at 250 mg/kg of concentration [110]. The methanolic seed extract of *L. sativum* also showed the highest antidiabetic activity against alloxan-induced albino rat at 300 mg/kg of dose [111].

### 5.4. Antioxidant

Plants are the major source of natural antioxidants, which function as free radical scavengers and reducing agents against reactive oxygen species and free radicals [112, 113].

The antioxidants present in the plant are found in the form of vitamins, phenols, terpenoids, flavonoids, coumarins, alkaloids, etc.

Researchers reported the antioxidant potential of *L. sativum* using different important antioxidants like gallic acid, coumarin acid, caffeic acid, quercetin, tocopherol (*α*, *β*, *γ*, *δ*), and among others [40, 41]. The ethanolic extract of stem, leaves, whole plant, and seeds of *L. sativum* was tested for antioxidant activity by several methods including 1,1-diphenyl-2-picrylhydrazyl (DPPH) scavenging assay, reduced glutathione assay, reducing power assay, and ascorbic acid content determination [37]. The result from the study suggests that all the parts (stem, leaves, whole plant, seeds) of *L. sativum* possess scavenging activity; however, the maximum per cent (12.19% ± 0.2) was noted for the whole plant, and the minimum per cent (2.69% ± 0.5) was noted for stem part. In reduced glutathione assay, all the extracts showed enhanced antioxidant activity; however, the highest value was measured in ethanolic leaf extract, i.e., 9 *μ*g/mL. Reducing power or Fe^3+^-Fe^2+^ transformation ability assay showed that all the plant parts possess the significant reducing ability [37].

Sat, Yildirim, Turan, and Demirbas [36] reported the antioxidant potential of species using DPPH assay in terms of EC_50_ value (EC_50_: 330.99 *μ*g/mL (Dadas, Turkey) and 346.65 *μ*g/mL (Dadas, Turkey) for FW and 128.08 and 85.97 *μ*g/mL for DW). However, Al-Saad and Al-Saadi [62] reported the IC_50_ value of 149.541 *μ*g/mL for the leaves of *L. sativum* by DPPH assay.

The DPPH, ABTS (2,2-azinobis (3-ethylbenzothiazoline-6-sulfonic acid)), FRAP (ferric [Fe(III)] ion–reducing antioxidant power), and *β*-carotene bleaching assays were performed to investigate the antioxidant activity of ethanolic and aqueous seed extract of *L. sativum*. The results showed 31.15% and 18.07% of DPPH activity and 24.61% and 21.14% ABTS activity for ethanolic and aqueous seed extracts, respectively. The FRAP and *β*-carotene/linoleic bleaching assay also confirm the antioxidant potential of the ethanolic and aqueous extract of *L. sativum* [42].

Kadam, Palamthodi, and Lele [40] also determined that the ethanolic seed extract of *L. sativum* possesses significant antioxidant activity using DPPH (IC_50_: 162.4 ± 2.3 *μ*g/mL), ABTS (IC_50_: 35.29 ± 1.02 *μ*g/mL), superoxide scavenging activity (IC_50_: 187.12 ± 3.4 *μ*g/mL), and metal chelating property (IC_50_: 119.32 ± 1.5 *μ*g/mL) assays [40].

Chatoui, Harhar, El Kamli, and Tabyaoui [38] evaluated the methanolic and ethanolic seed extracts from Tafraout, Rommani, Ben-Ahmed, and El-Haouz regions in Morocco for the estimation of antioxidant activity. The results showed that the highest DPPH (IC_50_: 119.3 *μ*g/mL), ABTS (IC_50_: 187.8 *μ*g/mL), and FRAP (EC_50_: 777.0 *μ*g/mL) activities in the methanolic seed extract of *L. sativum* are collected from Tafraout region [38]. Nitric oxide assay, total antioxidant capacity assay, reducing power assay, and hydrogen peroxide scavenging assay of aqueous and ethanolic seed extract of *L. sativum* showed the presence of significant antioxidant activity [43]. Few more studies from different regions confirm that the seed extract of *L. sativum* possesses significant amount of antioxidants and antioxidant activity [32, 39, 44, 74, 114, 115].

### 5.5. Antimicrobial

Presently, due to several environmental, biological, physical, chemical, and anthropogenic factors, the incidences of pathogenic microorganisms are increasing constantly, and this became a major concern among several scientific communities [116, 117]. The plant serves as a source of secondary metabolites which possess low or no side effects with other nutritional benefits. The antimicrobial activity of numerous medicinal plants has been studied against a range of microorganisms including bacteria, yeast, fungi, and virus, and many research groups are working continuously to discover novel antimicrobial compounds.

Hussain, Khattak, Muhammad, Khan, Khan, Ullah, and Haider [58] studied the antimicrobial activity of aqueous and chloroform plant extracts of *L. sativum* against a few bacterial strains including *Bacillus subtilis*, *Proteus vulgaris*, *Pseudomonas aeruginosa*, *Salmonella typhi*, *Staphylococcus aureus, Escherichia coli*, and two fungal strains*, Aspergillus niger* and *Candida albicans* by the agar well diffusion method [58]. The result showed that both the extracts possess antimicrobial activity against all the bacterial and fungal strains; however, the maximum and minimum zone of inhibition (ZI) for chloroform extract was shown by *P. vulgaris* (13 mm) and *S. typhi* (1 mm) and for aqueous extract, the maximum and minimum ZI was observed in *P. vulgaris* (16 mm) and *E. coli* (2 mm) [58].

The root, stem, and leaves were extracted with methanol, ethyl acetate, chloroform, and aqueous solvents and evaluated for antimicrobial activity. All extracts showed antimicrobial activity against bacterial strains (*E. coli*, *S. aureus*, *Klebsiella pneumoniae*, and *Salmonella typhimurium*) and fungal strains (*Aspergillus flavus*, *Aspergillus fumigatus*, *A. niger*, and *Fusarium solani*) [118]. Plant extract of *L. sativum* (ethanolic and aqueous) also showed antimicrobial activity against *Proteus* spp., *S. aureus*, and *Streptococcus* mutants by well diffusion method, whereas by minimum inhibitory concentrations (MIC), all the strains (*K. pneumoniae*, *Proteus spp., S. mutans*, *P. aeruginosa*, and *Staphylococcus aureus*) found to be sensitive to all concentrations (12.5%, 25%, 50%, 75%, and 100%) of the aqueous and ethanolic extracts of *L. sativum* [119].

The sprouts (dried and freeze dried) extract of the plant (*L. sativum*) is also examined for their antimicrobial activity against *K. pneumoniae*, *Proteus mirabilis*, *S. aureus*, *Salmonella infantis*, *S. epidermidis*, *E.coli*, and *P*. *aeruginosa* through well diffusion method [66]. Among dried and freeze-dried sprout extracts, the best result was observed in freeze-dried sprout extract showing maximum activity for *S. aureus* (21.5 mm), no activity was seen against *K. pneumoniae* and *E. coli*, and the MIC value for freeze-dried extract ranges between 0.5 and 1 mg/mL [66].

Ibrahim and Kebede [120] evaluated the antibacterial activities of aqueous and methanolic extracts of leaves of *L. sativum* against human pathogenic bacteria (*S. aureus*, *S. typhi*, *Streptococcus agalactiae*, *Shigella boydii*) [120]. Along with leaves, different seed extracts of *L. sativum* showed potential antimicrobial activity against a series of microbial strains (Table 6).

Gacemi et al. [121] reported the antifungal activity of lepidines B and E and compounds present in seeds of *L. sativum* against of *C. albicans*. The seed oil of *L. sativum* possesses antifungal and antibacterial activity against *S. aureus*, *B. subtilis*, *P.aeruginosa*, *E. coli*, *Salmonella enterica*, and *C. albicans.* The essential oil extracted by clevenger type apparatus from seeds of *L. sativum* showed the best activity at 1 mg/mL of concentration against *S. aureus* (15.57 ± 0.46 mm ZI), *B. cereus* (13.12 ± 1.16 mm ZI), *E. coli* (9.78 ± 065 mm ZI), and *K. pneumoniae* (8.17 ± 0.32 mm ZI) by disc diffusion assay [121].

### 5.6. Gastroprotective

Gastrointestinal infections are one of the most common problems in tropical countries. They involve various parts of the gastrointestinal tract and organs like the pancreas, liver and gallbladder [122]. They are responsible for causing problems like diarrhoea, abdominal distention, intestinal obstruction, abdominal pain, and gastrointestinal bleeding [123]. Gastrointestinal diseases directly or indirectly have an economic impact and also alter the quality of life Natural active compounds possess preventive and healing activity against gastrointestinal diseases [122, 124].

The methanolic extract of seed of *L. sativum* at 50, 100, and 200 mg/kg p.o. concentration was investigated for antidiarrheal activity against castor oil-induced diarrhoea in Swiss albino and Wistar rat models [125]. The highest antidiarrheal activity was observed in 200 mg/kg of concentration.

Rehman et al. [126] investigated the antidiarrheal and antispasmodic activities of seed extract of *L. sativum* against castor oil-induced diarrhoea in Sprague Dawley rat model at 100-300 mg/kg of doses [126]. The crude extract of seed was found to possess significant antidiarrheal and antispasmodic activity.

Another study by Mehmood et al. [127] investigated the aqueous-methanolic seed extract of *L. sativum* for indigestion and constipation at 30 and 100 mg/kg of doses in BALB/c mice, guinea pigs, and rabbits. The study showed the laxative and prokinetic effects of *L. sativum* seeds in the mice model [127].

### 5.7. Fracture/Bone Healing

Fracture healing or bone healing is a complicated physiological process that requires the participation of hematopoietic and immune cells in the bone marrow. Medicinal plants have important properties to reduce inflammation and pain of fractures and also help in fracture fast recovery [133, 134].

The impact of *L. sativum* seeds on fracture induced bone healing in rabbit (*Oryctolagus cuniculus*) model was evaluated. The test group had a statistically significant increase in the healing of fractures compared with the control group. The results showed the significant effect of *L. sativum* seeds in fracture induced bone healing [135]. Yadav et al. [136] reported the effect of ethanol seed extract (400 mg/kg p.o.) of *L. sativum* on fracture healing in the Wistar rat model.

The osteoprotective effect of *L. sativum* seeds (doses: 50-100 mg/kg) was studied in an ovary ectomized Wistar rat model [61]. Results revealed the antiosteoporotic actions of *L. sativum* with improved perpendicular and longitudinal femur compression strength.

Extract also enhanced the osteocalcin levels, and serum bone formation biomarkers lactate dehydrogenase (LDH) activity and inhibit the glutathione peroxidase (GPx) activity and deposition of lipid peroxides in bone tissues [61].

*L. sativum* showed a promising protective effect with no side effects against glucocorticoid-induced bone resorption in guinea pigs [137] and accelerates the alveolar bone healing and improves the formation of bone in periodontal diseases [138]. Alharbi et al. [139] investigated the in viv*o* effect of *L. sativum* seeds in osteogenic enhancement in bone fractures induced in *O. cuniculus* and concluded that the seeds can be used in the treatment of bone fractures [139].

## 6. The Challenge of Standardizing Extract, Toxicity, and Bioavailability of the Extract

Medicinal plants have shown immense pharmacological activities like fungicidal, bactericidal, virucidal, analgesic, anticancer, anti-inflammatory, neuroprotective, sedative, and antioxidant, due to the presence of significant phytochemicals or active compounds including flavonoids, phenols, terpenoids alkaloids, tannins, saponins, and glycosides [140–143].

Presently, excessive use of synthetic drugs and antibiotics has developed serious side effects, toxicity, and resistance against pathogenic microorganisms, which has limited their use in many countries; therefore, researchers are now paying more attention to traditional herbal medicines and their active compounds to fight against diseases and disorders [144–146].

One of the main clinical challenge is the reduced bioavailability and absorption of bioactive compounds from plants. As a result, their inclusion in nanoformulations with increased absorption, bioavailability, and transport to the target was the optimal therapeutic solution.

Bloukh et al. (2021) evaluate the antimicrobial potential of *Lepidium sativum* silver nanoparticles against a series of microbes by using agar well and disk diffusion assays. Pure extract and *Lepidium sativum* silver nanoparticle formulations displayed a significant antimicrobial activity (very good to intermediate) against 10 microbial strains (*S. pneumoniae, S. aureus, S. pyogenes, E. faecalis, B. subtilis, P. mirabilis, P. aeruginosa, E. coli, K. pneumoniae, C. albicans*) at the concentrations of 1.08 *μ*g/mL, 0.54 *μ*g/mL, and 0.27 *μ*g/mL [147].

Yasin et al. [148] evaluated the cytotoxicity of nanocapsulated lectin isolated from *L. sativum* against hepatocellular carcinoma cells (HepG2). The methanolic seed extract of *L. sativum* showed anticancer activity against in vivo Ehrlich ascite carcinoma (EAC) cell lines in Swiss albino mice at 500 mg/kg body weight of concentration [149].

*L. sativum* seed acetone extract and its combination with biogenic silver nanoparticles were found to be nontoxic to splenic cells [90].

## 7. Concluding Remarks

The current review discussed the traditional uses, nutritional values, chemical composition, and biological activity of *L. sativum*. Under this study, we summarized the presence of important minerals (potassium, calcium, phosphorus, iron, etc), amino acids (glutamic acid, leucine, etc.), fatty acid and essential oils (oleic acid, linoleic acid, linolenic acid, alpha-pinene, gamma-terpinene, alpha-terpineol, sabinene, alpha-phellandrene, etc.), and other secondary metabolites like campesterol, glucosinolates, napthalenol, furfural, coumarin, flavonoid, and phenolic acid in different extracts of *L. sativum.* The study also shows that it is an important edible herb that possesses wide range of therapeutic properties and high nutraceutical potential and can be used against malnutrition. However, most of the studies are restricted to in vitro studies and very few in vivo. Therefore, further research is needed to develop new phytopharmaceuticals based on *L. sativum*, and well-designed clinical studies are necessary to validate the biological activities reported in preclinical models mentioned in this review. Other than these scientific perspectives, people participation is needed regarding the planting, conservation, and sustainable use of *L. sativum* as a source of nutritionally rich food. Based on the scientific evidence, it can be concluded that *L. sativum* is a rich source of nutritional components along with bioactive compounds and could be used as a functional food.

## Figures and Tables

**Table 1:** Total phenolic and flavonoid content in *Lepidium sativum*.

Country	Plant part and solvents	Total phenolic content (mg gallic acid equivalent/g extract)	Total flavonoid content (mg quercetin equivalent/g extract)	Ref.
India	Ethanolic seed extract	4.46 ± 0.14	3.57 ± 1.2	[39]
	Ethanolic seed extract	11.03 ± 0.75	4.79 ± 0.24	[40]
Pakistan	Methanolic seed extract	120.26 ± 1.52^∗^	—	[41]
Egypt	Aqueous seed extract	126.24	007.21	[42]
	Ethanolic seed extract	88.08	00.65	[42]
	Ethanolic seed extract	46.00 ± 0.86	82.00 ± 0.93	[43]
	Aqueous seed extract	34.00 ± 0.67	53.00 ± 0.58	[43]
Turkey	Methanolic extract of aerial part	184.14 ± 2.5^∗∗^	12.63 ± 1.5^∗∗∗^	[44]
Morocco				
Tafraout region	Methanolic seed extract	94.48 ± 1.82	37.63 ± 2.14	[38]
	Ethanolic seed extract	86.48 ± 0.22	32.51 ± 0.81	[38]
El-Haouz region	Methanolic seed extract	83.36 ± 0.98	33.58 ± 0.33	[38]
	Ethanolic seed extract	80.28 ± 0.28	29.24 ± 0.47	[38]
Ben-Ahmed region	Methanolic seed extract	69.46 ± 0.09	24.85 ± 0.48	[38]
	Ethanolic seed extract	65.15 ± 1.07	23.92 ± 0.64	[38]
Rommani region	Methanolic seed extract	59.40 ± 0.62	21.09 ± 0.21	[38]
	Ethanolic seed extract	52.79 ± 0.30	20.04 ± 0.04	[38]

^∗^mg catechin equivalent/g extract; ^∗∗^*μ*g gallic acid equivalent/mg extract; ^∗∗∗^*μ*g quercetin equivalent/mg extract.

**Table 2:** The chemical composition of essential oils isolated from different parts of *L. sativum.*

Plant part used	Bioactive compounds	Regions/ country	References
Aerial part	Alpha-pinene; sabinene; limonene; 1,8-cineole; *β*-thujone; phenylacetonitrile; benzyl isothiocyanate; hexadecanoic acid; linoleic acid.	Iran	[45]

Seeds	Alpha-pinene; 1,8-cineole; phenylacetonitrile; benzyl isothiocyanate.	Iran	[45]
	Alpha-pinene; sabinene; alpha-phellandrene; eucalyptol; gamma-terpinene; linanool; terpinen-4-ol; alpha-terpineol; propanoate; alpha-terpinyl acetate; E-nerolidol.	Greece	[49]
	Docosatrienoic acid; linoleic acid; eicosenoic acid; palmitic acid; arachidonoic acid; eruic acid; stearic acid; arachidic acid.	Ethiopia	[50]
	Alpha-tocopherol; gamma-tocopherol; cholesterol; campesterol; stigmasterol; sitosterol; avenasterol.	USA	[47]
	7,10-Hexadecadienoic acid, methyl ester; 11-octadecenoic acid, methyl ester; behenic acid, methyl ester; 7,10,13-hexadecatrienoic acid, methyl ester; stearic acid, methyl ester; hexadecanoic acid, 15-methyl-, methyl ester; 15-tetracosenoic acid, methyl ester; 10-octadecenoic acid, methyl ester; heneicosanoic acid, methyl ester.	Saudi Arabia	[51]
	Myristic acid; palmitic acid; palmitoleic acid; stearic acid; oleic acid; linoleic acid; linolenic acid; arachidic acid; gadoleic acid; cholesterol acid; stigmasterol; campesterol; beta-sitosterol; 5-avenasterol; alpha-tocopherol; beta-tocopherol; gamma-tocopherol.	Morocco	[38]
	Toluene, octane, (E,Z)-1,3,5-octatriene, ethylbenzene, 2-furanmethanol, styrene, methional, benzaldehyde, benzyl alcohol, benzaldehyde, benzyl alcohol, 1-isocyano-2-methylbenzene, benzyl isothiocyanate, benzylurea, 1-benzyl-2(1 H)-pyridone, (Z)-8-heptadecene, palmitic acid, cyclic octaatomic sulfur, oleic acid, linoleic acid, nonadecanamide, arachidic acid, etc.	India	[20]
	Linolenic acid, oleic acid, arachidic acid, palmitic acid, stearic acid.	India	[52]
	Myristic acid; palmitoleic acid; palmitic acid; alpha-linolenic acid; octadecenoic acid; stearic acid; 9-octadecen-12-ynoic acid; paullinic acid; arachidic acid; erucic acid; behenic acid; nervonic acid; lignoceric acid.	Saudi Arabia	[53]
	Alpha-linolenic acid; oleic acid; linoleic acid; eicosanoic acid; palmitic acid; erucic acid; arachidic acid; stearic acids.	India	[46]
	Beta-amyrin; 9,12,15-octadecatrienoic acid methyl ester; 9-octadecenoic acid methyl ester; alpha-amyrin; 11-eicosenoic acid methyl ester; 9,12-octadecadienoic acid; hexadecanoic acid methyl ester.	Saudi Arabia	[54]
	Geraniol; citronellol; nerol; triacontane; palmitic acid; 1,6-octadien-3-ol, 3,7-dimethyl.	Egypt	[55]

Roots	Alpha-pinene; benzyl isothiocyanate; hexadecanoic acid; linoleic acid.	Iran	[45]

**Table 3:** The chemical composition of *Lepidium sativum* extracts.

Plant part used	Bioactive compounds	Regions/country	References
Leaves	Benzyl nitrile n,n-Dimethylaminoethanol 2-Hydroxy-1-(1′-pyrrolidiyl)-1-buten-3-one d-Proline Butyrolactone	Iraq	[62]
	Apigenin Quercetin Kaempferol Luteolin 7-Hydroxy-4′,5,6-trimethoxyisoflavone; Sinapic acid Chlorogenic acid p-coumaric acid Ascorbic acid *α*-Tocopherol 6-prenylnaringenin.	Egypt	[63]

Seeds	Glucotropaeolin; sinapine K di-hexose rhamnose Sinapoyl di-glucose; sinapoyl malate K hexose rhamnose 1 K rhamnose (benzo) di-hexose 1	Algeria	[64]
	Benzyl nitrile Benzene-isothiocyanatomethyl 3′,5′-dimethoxyacetophenone Hexadecanoic acid methyl ester cis-Vaccenic acid cs-11-Eicosenoic acid-methyl ester 7,8-Epoxylanostan-11-ol, 3-acetoxyeergosta-14,22-dien-3-ol- acetate -3 beta-5 alpha	India	[65]
	Benzyl cyanide Benzyl thiocyanate Benzyl isothiocyanate Benzaldehyde Benzonitrile Benzyl thiocyanate Benzyl isothiocyanate	Poland	[66]

Aerial part	Stigmast-5-en-3 *β*27-Diol 27-benzoate	India	[67]

**(a):** 

Proximate composition				
Component	Nigeria (g/100 g DW ± SD) [68]	Bangladesh (g/100 g DW ± SD) [71]	Nigeria (%) [72]
Moisture	91.05 ± 1.41	87.13 ± 0.088	81.85
Ash	15.38 ± 0.21	1.80 ± 0.015	3.25
Crude fiber	9.31 ± 0.13	2.38 ± 0.015	8.69
Crude protein	18.25 ± 0.1	2.53 ± 0.041	1.01
Total carbohydrate	55.34 ± 0.20	5.47 ± 0.025	5.82
Total lipid	1.72 ± 0.18	—	8.08
Total fat	—	0.70 ± 0.029	—

**(b):** 

Minerals		
Principal component	Nigeria (mg/100 g DW ± SD) [68]
Potassium	1850.00 ± 43.30
Phosphorus	4.10 ± 0.44
Magnesium	160.60 ± 6.56
Calcium	829.13 ± 20.70
Iron	63.47 ± 5.27
Sodium	141.13 ± 38.19
Copper	0.39 ± 0.02
Chromium	0.36 ± 0.27
Zinc	2.28 ± 0.07
Manganese	5.74 ± 0.11

**(c):** 

Aminoacids		
Principal component	Nigeria (g/100 g protein DW ± SD) [68]
Isoleucine (Ile)^∗^	3.26 ± 1.05
Leucine (Leu)^∗^	6.84 ± 1.02
Lysine (Lys)^∗^	3.5 ± 0.21
Methionine (Met)^∗^	1.11 ± 0.1
Cysteine (Cys)	0.42 ± 0.21
Phenylalanine (Phe)^∗^	4.77 ± 2.02
Tyrosine (Tyr)	2.59 ± 1.20
Threonine (Thr)^∗^	2.61 ± 1.04
Valine (Val)^∗^	3.85 ± 0.25
Alanine (Ala)	4.31 ± 0.90
Arginine (Arg)^∗^	4.32 ± 1.78
Aspartic acid (Asp)	7.73 ± 2.77
Glutamic acid (Glu)	9.36 ± 0.06
Glycine (Gly)	1.24 ± 0.24
Histidine (His)^∗^	2.09 ± 1.00
Proline (Pro)	2.16 ± 0.16
Serine (Ser)	2.31 ± 0.01

^∗^Essential amino acids. DW: dry weight; SD: standard deviation.

**(a):** 

Proximate content					
Component	India (g/100 g) [70]	Indian (g/100 g DW) [73]	Saudi Arabia (%) [69]	Pakistan (%) [41]	Egypt (%) [74]
Moisture	4.14 ± 0.05	4.82 ± 0.09	4.89 ± 0.050	3.92 ± 1.06	7.05 ± 0.45
Ash	4.65 ± 0.09	4.95 ± 0.00	5.83 ± 0.389	4.25 ± 0.13	4.8 ± 0.88
Crude fiber	7.01 ± 0.08	9.72 ± 0.32	6.80 ± 0.080	6.75 ± 1.02	18.79 ± 0.79
Crude protein	22.47 ± 0.78	26.31 ± 0.03	19.82 ± 0.205	24.18 ± 1.5	19.73 ± 1.03
Total carbohydrate	34.24 ± 0.92	29.25 ± 0.27	34.24 ± 0.092	32.87 ± 0.29	35.45 ± 1.65
Total lipid	—	—	—	28.03 ± 1.05	—
Total fat	27.48 ± 0.14	24.96 ± 0.02	—	—	14.18 ± 0.94

**(b):** 

Mineral composition			
Principal component	India (mg/100 g ± SD) [70]	Saudi Arabia (mg/100 g ± SD) [69]	Pakistan (mg/100 g of seed ± SD) [41]
Potassium	1193.95 ± 10.51	785.0 ± 7.51	1236.51 ± 1.67
Phosphorus	514.59 ± 10.67	616.50 ± 9.67	608.63 ± 1.39
Magnesium	315.25 ± 3.63	—	339.23 ± 2.13
Calcium	296.60 ± 1.04	253.0 ± 1.04	266.35 ± 1.44
Iron	7.62 ± 0.04	53.81 ± 0.04	8.31 ± 0.36
Sodium	24.64 ± 0.02	—	19.65 ± 0.98
Copper	5.53 ± 0.09	1.90 ± 0.09	5.73 ± 2.11
Zinc	5.05 ± 0.07	4.10 ± 0.07	6.99 ± 0.54
Manganese	2.57 ± 0.04	—	2.00 ± 1.08
Sulphur	293.02 ± 14.27	—	—
Aluminum	2.82 ± 0.13	—	—
Boron	1.41 ± 0.03	—	—
Molybdenum	0.43 ± 0.08	—	—

**(c):** 

Fatty acid profile			
Fatty acid	India (%) [70]	Saudi Arabia (%) [69]	Pakistan (%) (g/100 g of *L*.*sativum*) ± SD [41]
Palmitic acid	8.7	8.80	10.30 ± 0.12
Oleic acid	19.9	23.49	30.50 ± 0.16
Palmitoleic acid	—	—	0.70 ± 0.30
Stearic acid	3.2	3.49	1.90 ± 0.19
Myristic acid	1.9	1.50	—
Linolenic acid	12.1	30.07	—
Linoleic acid	30.2	11.35	8.60 ± 0.38
Eicosenoic acid	10.3	12.60	—
Erucic acid	—	4.64	—
Arachidic acid	3.2	4.06	—

**(d):** 

Amino acid composition		
Principal component	Saudi Arabia (g/100 g protein ± SD) [69]	Pakistan (g/100 g protein ± SD) [70]
Isoleucine (Ile)^∗^	5.21 ± 0.014	5.11 ± 0.03
Leucine (Leu)^∗^	9.03 ± 0.007	8.21 ± 0.01
Lysine (Lys)^∗^	2.26 ± 0.390	6.26 ± 0.39
Methionine (Met)^∗^	1.86 ± 0.000	0.97 ± 0.02
Cysteine (Cys)	0.80 ± 0.000	—
Phenylalanine (Phe)^∗^	5.80 ± 0.004	5.65 ± 0.03
Tyrosine (Tyr)	3.82 ± 0.000	2.69 ± 0.09
Threonine (Thr)^∗^	5.39 ± 0.019	2.66 ± 0.09
Valine (Val)^∗^	6.24 ± 0.007	8.04 ± 0.03
Alanine (Ala)	—	4.83 ± 0.02
Arginine (Arg)^∗^	—	4.51 ± 0.03
Aspartic acid (Asp)	—	9.76 ± 0.03
Glutamic acid (Glu)	—	19.33 ± 0.19
Glycine (Gly)	—	5.51 ± 0.07
Histidine (His)^∗^	3.51 ± 0.007	3.87 ± 0.14
Proline (Pro)	—	5.84 ± 0.38
Serine (Ser)	—	4.96 ± 0.09
Phenylalanine + tyrosine (Phe + Tyr)	9.62 ± 0.000	—
Methionine + cysteine (Met + Cys)	1.86 ± 0.000	—

^∗^Essential amino acids. DW: dry weight; SD: standard deviation.

**Table 6:** Antimicrobial activities of different extracts of *L. sativum.*

Extract/concentration	Control drug used	Microorganisms	Agar well diffusion method/agar disc diffusion method ZI (mm); control drug (ZI)	MIC/MBC (mg/mL)	Regions	References
Chloroform extract (100 mg/mL)	Gentamicin	*Escherichia coli* *Salmonella typhi* *Pseudomonas aeruginosa* *Staphylococcus aureus* *Bacillus cereus* *Micrococcus luteus*	NZ; 22 NZ; 24 NZ; 21 10; 25 NZ; 28 11; 27	NT	India	[128]
Ethyl acetate extract (100 mg/mL)	Gentamicin	*Escherichia coli* *Salmonella typhi* *Pseudomonas aeruginosa* *Staphylococcus aureus* *Bacillus cereus* *Micrococcus luteus*	14; 22 NZ; 24 NZ; 21 18; 25 NZ; 28 16; 27	NT		
Methanol extract (100 mg/mL)	Gentamicin	*Escherichia coli* *Salmonella typhi* *Pseudomonas aeruginosa* *Staphylococcus aureus* *Bacillus cereus* *Micrococcus luteus*	14; 22 13; 24 14; 21 22; 25 16; 28 16; 27	6.25/25 25/ND 6.25/25 1.56/6.25 6.25/25.0 12.5/ND		
Dichloromethane extract (100 mg/mL)	Gentamicin	*Escherichia coli* *Salmonella typhi* *Pseudomonas aeruginosa* *Staphylococcus aureus* *Bacillus cereus* *Micrococcus luteus*	NZ; 22 NZ; 24 NZ; 21 NZ; 25 NZ; 28 10; 27	NT		
Petroleum ether extracts (2.5%)	Gentamicin/ Ketoconzol	*Staphylococcus aureus* *Escherichia coli* *Klebsiella pneumoniae* *Proteus vulgaris* *Pseudomonas aeruginosa* *Candida albicans*	25; 32 25; 32 26; 35 21; 34 18; 32 32; 33	NT	Sudan	[129]
Methanolic extract (2.5%)	Gentamicin/Ketoconzol	*Staphylococcus aureus* *Escherichia coli* *Klebsiella pneumoniae* *Proteus vulgaris* *Pseudomonas aeruginosa* *Candida albicans*	15; 32 17; 32 16; 35 18; 34 17; 32 9; 33			
Aqueous extract (10%)	Gentamicin/ Ketoconzol	*Staphylococcus aureus* *Escherichia coli* *Klebsiella pneumoniae* *Proteus vulgaris* *Pseudomonas aeruginosa* *Candida albicans*	NZ; 32 19; 32 17; 35 19; 34 16; 32 21; 33			
Ethanolic extract (1 mg/mL)	Not mentioned	*Staphylococcus aureus* *Listeria monocytogenes* *Salmonella Enteritidis* *Escherichia coli* *Serratia marcescens*	10∗ 10∗ 20∗ 12∗ 7	NT	Egypt	[54]
Methanol extract (1 mg/mL)	Not mentioned	*Salmonella Enteritidis* *Serratia marcescens*	15 9			
Methanol extract (100 mg/mL)	Gentamicin/ nystatin	*Escherichia coli* *Staphylococcus aureus* *Bacillus subtilis* *Candida albicans* *Aspergillus niger*	14; 32 13; 35 13; 29 14; 17 20; 20	NT	Sudan	[130]
Ethanol extract (50 mg/mL)	Vancomycin	*Escherichia coli* *Pseudomonas aeruginosa*	22.63; 18 10; NA	NT	Ethiopia	[131]
Methanol extract (50 mg/mL)	Vancomycin	*Escherichia coli* *Pseudomonas aeruginosa*	22.37; 18 9; NA			
Chloroform extract (50 mg/mL)	Vancomycin	*Escherichia coli* *Pseudomonas aeruginosa*	10.67; 18 11.33; NA			
Ethyl acetate extract	Erythromycin	*Rhodococcus equi*	15.5; 23	NT	Morocco	[18]
Methanolic extract	Chlorophénicol	*Rhodococcus equi*	13.15; 34			
Petroleum ether extract	Ciprofloxacine	*Rhodococcus equi*	10.35; 30			
Ethanol extract	Not mentioned	*Pseudomonas aeruginosa* *Klebsiella pneumonia* *Escherichia coli* *Staphylococcus aureus* *Streptococcus pyogenes* M*β*L, *P. aeruginosa* ES*β*L, *E. coli* MRSA MDR *S. pyogenes*	NT	12.5/25 6.25/12.5 3.13/3.13 6.25/6.25 50/50 25/25 12.5/12.5 12.5/25 100/50	Egypt	[132]
Acetone extract	Not mentioned	*Pseudomonas aeruginosa* *Klebsiella pneumonia* *Escherichia coli* *Staphylococcus aureus* M*β*L, *P. aeruginosa* ES*β*L, *Klebsiella pneumonia* ES*β*L, *E. coli* MRSA		25/25 12.5/25 6.25/12.5 3.13/6.25 25/50 12.5/12.5 12.5/25 12.5/25		
Aqueous extract	Not mentioned	*Pseudomonas aeruginosa* *Escherichia coli* *Staphylococcus aureus* *E. coli* MRSA		25/25 6.25/6.25 6.25/12.5 12.5/25		
n-Butanol extract	Not mentioned	*Escherichia coli* *Pseudomonas aeruginosa* *Staphylococcus aureus* (methicillin-sen.) *Staphylococcus aureus* (methicillin-res.) *Enterococcus faecalis*	NT	5 4 4 4 3.5	Algeria	[64]

Abbreviations: NZ: no zone of inhibition; NA: not applicable; NT: not tested; MBC: minimum bactericidal concentration; MIC: minimum inhibitory concentration.

## Data Availability

The data supporting this review are from previously reported studies and datasets, which have been cited. The processed data are available from the corresponding author upon request.

## References

[1] Semwal P., Painuli S., Cruz-Martins N. (2021). *Dioscorea deltoidea* wall. Ex Griseb: A review of traditional uses, bioactive compounds and biological activities. *Food Bioscience*.

[2] Semwal P., Painuli S., Tewari D., Bussmann R. W., Palni L. M. S., Thapliyal A. (2020). Assesment of non-timber Brahma Kamal (*Saussurea obvallata* (DC.) Edgew.), an important Himalayan medicinal plant: Ethnomedicinal, phytochemical and pharmacological overview. *Ethnobotany Research and Applications*.

[3] Balakrishnan R., Vijayraja D., Jo S. H., Ganesan P., Su-Kim I., Choi D. K. (2020). Medicinal profile, phytochemistry, and pharmacological activities of *Murraya koenigii* and its primary bioactive compounds. *Antioxidants (Basel)*.

[4] Ujowundu C., Okafor O., Agha N., Nwaogu L., Igwe K., Igwe C. (2010). Phytochemical and chemical composition of *Combretum zenkeri* leaves. *Journal of Medicinal Plant Research*.

[5] Kala C. P., Dhyani P. P., Sajwan B. S. (2006). Developing the medicinal plants sector in northern India: challenges and opportunities. *Journal of Ethnobiology and Ethnomedicine*.

[6] Aqafarini A., Lotfi M., Norouzi M., Karimzadeh G. (2019). Induction of tetraploidy in garden cress: morphological and cytological changes. *Plant Cell, Tissue and Organ Culture (PCTOC)*.

[7] Bansal D., Bhasin P., Yadav O., Punia A. (2012). Assessment of genetic diversity in *Lepidium sativum* (Chandrasur) a medicinal herb used in folklore remedies in India using RAPD. *Journal, Genetic Engineering & Biotechnology*.

[8] Roughani A., Miri S. M. *Lepidium* species as antidiabetic herbal medicines. *Proceedings of The First National Congress and International Fair of Medicinal Plants and Strategies for Persian Medicine that Affect Diabetes*.

[9] Kiple K. F., Ornelas K. (2000). *The Cambridge World History of Food*.

[10] Hadi M. Y., Hameed I. H. (2017). Uses of gas chromatography-mass spectrometry (GC-MS) technique for analysis of bioactive chemical compounds of *Lepidium sativum*: a review. *Research Journal of Pharmacy and Technology*.

[11] Batsatsashvili K., Kikvidze Z., Bussmann R. (2019). *Ethnobotany of the Mountain Regions of Far Eastern Europe*.

[12] Sokolov P. (1985). *Rastitelnye Resursy SSSR: Tsvetkovye Rasteniia, Ikh Khimicheskii Sostav, Ispolzovanie. Semeistva Paeoniaceae—Thymelaeaceae [Plant Resources of the USSR: Flowering Plants, their Chemical Composition, Utilization; Family Paeoniaceae—Thymelaeaceae]*.

[13] Hussein H. J., Hameed I. H., Hadi M. Y. (2017). Using gas chromatography-mass spectrometry (GC-MS) technique for analysis of bioactive compounds of methanolic leaves extract of *Lepidium sativum*. *Research Journal of Pharmacy and Technology*.

[14] Grossheim A. (1952). *Plant Richness of the Caucasus*.

[15] Sokolov P. (1990). *Plant Resources of the USSR. Flowering Plants, their Chemical Composition and Use. L*.

[16] Prajapati V. D., Maheriya P. M., Jani G. K., Patil P. D., Patel B. N. (2014). *Lepidium sativum* Linn.: a current addition to the family of mucilage and its applications. *International Journal of Biological Macromolecules*.

[17] Agarwal J., Verma D. (2011). Antioxidative activity and flavonoid composition from *Lepidium sativum*. *Natural Science*.

[18] Chatoui K., Talbaoui A., Aneb M., Bakri Y., Harhar H., Tabyaoui M. (2016). Phytochemical screening, antioxidant and antibacterial activity of *Lepidium sativum* seeds from Morocco. *J Mater Environ Sci*.

[19] Eddouks M., Maghrani M., Zeggwagh N. A., Michel J. B. (2005). Study of the hypoglycaemic activity of *Lepidium sativum* L. aqueous extract in normal and diabetic rats. *Journal of Ethnopharmacology*.

[20] Getahun T., Sharma V., Gupta N. (2020). Chemical composition, antibacterial and antioxidant activities of oils obtained by different extraction methods from *Lepidium sativum* L. seeds. *Industrial Crops and Products*.

[21] Raish M., Ahmad A., Alkharfy K. M. (2016). Hepatoprotective activity of *Lepidium sativum* seeds against D-galactosamine/lipopolysaccharide induced hepatotoxicity in animal model. *BMC Complementary and Alternative Medicine*.

[22] PlantList T. http://www.theplantlist.org/.

[23] Heinrich M., Appendino G., Efferth T. (2020). Best practice in research – overcoming common challenges in phytopharmacological research. *Journal of Ethnopharmacology*.

[24] Sharifi-Rad J., Quispe C., Rahavian A. (2021). Bioactive compounds as potential agents for sexually transmitted diseases management: a review to explore molecular mechanisms of action. *Frontiers in Pharmacology*.

[25] Sharifi-Rad J., Bahukhandi A., Dhyani P. (2021). Therapeutic potential of neoechinulins and their derivatives: an overview of the molecular mechanisms behind pharmacological activities. *Frontiers in Nutrition*.

[26] Sharifi-Rad J., Quispe C., Shaheen S. (2021). Flavonoids as potential anti-platelet aggregation agents: from biochemistry to health promoting abilities. *Critical Reviews in Food Science and Nutrition*.

[27] Salehi B., Quispe C., Chamkhi I. (2021). Pharmacological properties of Chalcones: a review of preclinical including molecular mechanisms and clinical evidence. *Frontiers in Pharmacology*.

[28] Sharifi-Rad J., Kamiloglu S., Yeskaliyeva B. (2020). Pharmacological activities of psoralidin: a comprehensive review of the molecular mechanisms of action. *Frontiers in Pharmacology*.

[29] Salehi B., Sharifi-Rad J., Cappellini F. (2020). The therapeutic potential of anthocyanins: current approaches based on their molecular mechanism of action. *Frontiers in Pharmacology*.

[30] Al-Yahya M. A., Mossa J. S., Ageel A. M., Rafatullah S. (1994). Pharmacological and safety evaluation studies on *Lepidium sativum* L., Seeds. *Phytomedicine*.

[31] Fan Q. L., Zhu Y. D., Huang W. H., Qi Y., Guo B. L. (2014). Two new acylated flavonol glycosides from the seeds of *Lepidium sativum*. *Molecules*.

[32] Kumar V., Tomar V., Ranade S., Yadav H., Srivastava M. (2020). Phytochemical, antioxidant investigations and fatty acid composition of *Lepidium sativum* seeds. *Journal of Environmental Biology*.

[33] Singh C. S., Paswan V. K. (2017). The potential of garden cress (*Lepidium sativum* L.) seeds for development of functional foods. *Advances in Seed Biology*.

[34] Mazumder A., Dwivedi A., Du Plessis J. (2016). Sinigrin and its therapeutic benefits. *Molecules*.

[35] Barba F. J., Nikmaram N., Roohinejad S., Khelfa A., Zhu Z., Koubaa M. (2016). Bioavailability of glucosinolates and their breakdown products: impact of processing. *Frontiers in Nutrition*.

[36] Sat I. G., Yildirim E., Turan M., Demirbas M. (2013). Antioxidant and nutritional characteristics of garden cress (*Lepidium sativum*). *Acta Sci. Polonorum-Hort. Cultus*.

[37] Malar J., Chairman K., Singh A. R. J., Vanmathi J. S., Balasubramanian A., Vasanthi K. (2014). Antioxidative activity of different parts of the plant *Lepidium sativum* Linn. *Biotechnology Reports*.

[38] Chatoui K., Harhar H., El Kamli T., Tabyaoui M. (2020). Chemical composition and antioxidant capacity of *Lepidium sativum* seeds from four regions of Morocco. *Evidence-based Complementary and Alternative Medicine*.

[39] Yadav Y., Srivastav D., Saini V. (2011). In vitro antioxidant activities of ethanolic extract of *Lepidium sativum* L. *Seeds. An International Journal of Pharmaceutical Sciences*.

[40] Kadam D., Palamthodi S., Lele S. S. (2018). LC-ESI-Q-TOF-MS/MS profiling and antioxidant activity of phenolics from L. *Sativum seedcake. J Food Sci Technol*.

[41] Zia-Ul-Haq M., Ahmad S., Calani L. (2012). Compositional study and antioxidant potential of *Ipomoea hederacea* Jacq. and *Lepidium sativum* L. seeds. *Molecules*.

[42] El-Maati M. F. A., Labib S. M., Al-Gaby A. M., Ramadan M. F. (2016). Antioxidant and antibacterial properties of different extracts of garden cress (*Lepidium sativum* L.). *Zagazig Journal of Agricultural Biochemistry and its Application*.

[43] Abdulmalek S. A., Fessal M., El-Sayed M. (2021). Effective amelioration of hepatic inflammation and insulin response in high fat diet-fed rats via regulating AKT/mTOR signaling: role of *Lepidium sativum* seed extracts. *Journal of Ethnopharmacology*.

[44] Selek S., Koyuncu I., Caglar H. G. (2018). The evaluation of antioxidant and anticancer effects of *Lepidium Sativum* Subsp Spinescens L. methanol extract on cancer cells. *Cellular and Molecular Biology (Noisy-le-Grand, France)*.

[45] Afsharypuor S., Hadi M. E.'. (2006). Volatile constituents of the seeds, roots and non-flowering aerial parts of *Lepidium satvium* L. *Journal of Essential Oil Research*.

[46] Diwakar B. T., Dutta P. K., Lokesh B. R., Naidu K. A. (2009). Physicochemical properties of garden cress (*Lepidium sativum* L.) seed oil. *Journal of the American Oil Chemists' Society*.

[47] Moser B. R., Shah S. N., Winkler-Moser J. K., Vaughn S. F., Evangelista R. L. (2009). Composition and physical properties of cress (*Lepidium sativum* L.) and field pennycress (*Thlaspi arvense* L) oils. *Industrial Crops and Products*.

[48] Singh B., Jain D., Joshi A. (2020). Molecular diversity analysis and metabolic profiling of seed oil in *Lepidium sativum* L. Genotypes. *Genotypes. Plant Molecular Biology Reporter*.

[49] Kimbaris A. C., Koliopoulos G., Michaelakis A., Konstantopoulou M. A. (2012). Bioactivity of *Dianthus caryophyllus*, *Lepidium sativum*, *Pimpinella anisum*, and *Illicium verum* essential oils and their major components against the West Nile vector Culex pipiens. *Parasitology Research*.

[50] Solomon G., Aman D., Bachheti R. (2016). Fatty acids, metal composition, nutritional value and physicochemical parameters of *Lepidium sativium* seed oil collected from Ethiopia. *International Food Research Journal*.

[51] Alqahtani F. Y., Aleanizy F. S., Mahmoud A. Z. (2019). Chemical composition and antimicrobial, antioxidant, and anti-inflammatory activities of *Lepidium sativum* seed oil. *Saudi journal of biological sciences*.

[52] Kharkwal N., Prasad R., Kumar S. (2021). Physico-chemical characterisation of *Lepidium sativum* (garden cress) GA-1 seed. *Journal of Pharmacognosy and Phytochemistry*.

[53] Al-Asmari A. K., Athar M. T., Al-Shahrani H. M., Al-Dakheel S. I., Al-Ghamdi M. A. (2015). Efficacy of *Lepidium sativum* against carbon tetra chloride induced hepatotoxicity and determination of its bioactive compounds by GC ⿿MS. *Toxicology Reports*.

[54] Abo El-Maati M. F., Labib S. M., Al-Gaby A., Ramadan M. F. (2016). Antioxidant and antibacterial properties of different extracts of garden cress (*Lepidium sativum* L.). Zagazig. *Journal of Agricultural Research*.

[55] Kamel R., Elmotasem H., Abdelsalam E., Salama A. (2021). *Lepidium sativum* seed oil 3D nano-oleogel for the management of diabetic wounds: GC/MS analysis, *in-vitro* and **in-vivo** studies. *Journal of Drug Delivery Science and Technology*.

[56] Radwan H., El-Missiry M., Al-Said W., Ismail A., Abdel Shafeek K., Seif-El-Nasr M. (2007). Investigation of the glucosinolates of *Lepidium sativum* growing in Egypt and their biological activity. *Res J Med Med Sci*.

[57] Williams D. J., Critchley C., Pun S., Chaliha M., O’Hare T. J. (2009). Differing mechanisms of simple nitrile formation on glucosinolate degradation in *Lepidium sativum* and *Nasturtium officinale* seeds. *Phytochemistry*.

[58] Hussain I., Khattak M. U. R., Muhammad Z. (2011). Phytochemicals screening and antimicrobial activities of selected medicinal plants of Khyberpakhtunkhwa Pakistan. *African Journal of Pharmacy and Pharmacology*.

[59] Maier U. H., Gundlach H., Zenk M. H. (1998). Seven imidazole alkaloids from *Lepidium sativum*. *Phytochemistry*.

[60] El-Gendy R. (2021). Toxicological, histological and biochemical effects of *Lepidium sativum* seeds extract on *Galleria mellonella* L. (Lepidoptera: Pyralidae) larvae. *Catrina: The International Journal of Environmental Sciences*.

[61] Abdallah H. M., Farag M. A., Algandaby M. M. (2020). Osteoprotective activity and metabolite fingerprint via UPLC/MS and GC/MS of *Lepidium sativum* in Ovariectomized rats. *Nutrients*.

[62] Al-Saad O. A., Al-Saadi S. A. M. (2021). Chemical composition and antioxidants of *Lepidium Sativum* and L. aucheri. *University of Thi-Qar Journal of Science*.

[63] El-Haggar M., El-Hosseiny L., Ghazy N. M., El-Fiky F. K., El-Hawiet A. (2021). Phytochemical investigation, antimicrobial and cytotoxic activities of suspension cultures of *Lepidium sativum* L.. *South African Journal of Botany*.

[64] Ait-Yahia O., Perreau F., Bouzroura S.-A., Benmalek Y., Dob T., Belkebir A. (2018). Chemical composition and biological activities of n-butanol extract of *Lepidium sativum* L (Brassicaceae) seed. *Tropical Journal of Pharmaceutical Research*.

[65] Rajasekaran R., Suresh P. (2021). Physical and chemical methods of extraction of bioactive molecules from *Lepidium sativum* Linn. And antioxidant activity-based screening and selection of extracts-probable phytochemical, chromatography and mass spectroscopy analysis-based correlates. *Research Journal of Pharmacy and Technology*.

[66] Rafińska K., Pomastowski P., Rudnicka J. (2019). Effect of solvent and extraction technique on composition and biological activity of *Lepidium sativum* extracts. *Food Chemistry*.

[67] Mughal M. H., Ali M., Iqbal M., Srivastava P. (1999). A steryl ester from *Lepidium sativum*. *Phytochemistry*.

[68] Hassan L. G., Hassan S. W., Hashim T., Umar K. J., Sani N. A. (2012). Determination of nutritive values of garden cress (*Lepidium Sativum* L.) leaves. *Bayero Journal of Pure and Applied Sciences*.

[69] Alshammari G. M., Yahya M. A., Ahmed S. B. (2017). Nutritive value of Elrashad (*Lepidium sativum* L.) seeds grown in Saudi Arabia. *Journal of Experimental Biology and Agricultural Sciences*.

[70] Gokavi S. S., Malleshi N. G., Guo M. (2004). Chemical composition of garden cress (*Lepidium sativum*) seeds and its fractions and use of bran as a functional ingredient. *Plant foods for human nutrition (Dordrecht, Netherlands)*.

[71] Hossain M. A., Rahman M. A., Sazia S., Sakil M. A., Polash M. A. S. (2020). Production time and nutritional assessment of garden cress (*Lepidium sativum* L.) leaves for ethno-botanical uses in Bangladesh. *Asian Journal of Advances in Agricultural Research*.

[72] Umar A., Jimoh W. L. O., Garba M. D. (2011). Proximate analysis of *Lepidium sativum* leaves. *ChemSearch Journal*.

[73] Choudhary P., Gupta R., Verma R. (2019). Impact of dietary and nutrition education intervention on nutritional knowledge of moderately anemic adolescent girls of kangra district, Himachal Pradesh. *Indian Journals.com*.

[74] El-Salam A., Kholoud H., Toliba A., El-Shourbagy G. A., El-Nemr S. E. (2019). Chemical and functional properties of garden cress (*Lepidium sativum* L.) seeds powder. Zagazig. *Journal of Agricultural Research*.

[75] Salehi B., Calina D., Docea A. O. (2020). Curcumin's nanomedicine formulations for therapeutic application in neurological diseases. *Journal of Clinical Medicine*.

[76] Sharifi-Rad J., Quispe C., Butnariu M. (2021). Chitosan nanoparticles as a promising tool in nanomedicine with particular emphasis on oncological treatment. *Cancer Cell International*.

[77] Zlatian O. M., Comanescu M. V., Rosu A. F. (2015). Histochemical and immunohistochemical evidence of tumor heterogeneity in colorectal cancer. *Romanian Journal of Morphology and Embryology*.

[78] Sani T. A., Mohammadpour E., Mohammadi A. (2017). Cytotoxic and apoptogenic properties of Dracocephalum kotschyi aerial part different fractions on CALU-6 and MEHR-80 lung cancer cell lines. *Farmácia*.

[79] Salehi B., Lopez-Jornet P., Pons-Fuster López E. (2019). Plant-derived bioactives in oral mucosal lesions: a key emphasis to curcumin, lycopene, chamomile, Aloe vera Green Tea and Coffee Properties. *Green Tea and Coffee Properties. Biomolecules*.

[80] Salehi B., Prakash Mishra A., Nigam M. (2021). *Ficus* plants: state of the art from a phytochemical, pharmacological, and toxicological perspective. *Phytotherapy Research*.

[81] Salehi B., Shivaprasad Shetty M., V Anil Kumar N. (2019). *Veronica* plants-drifting from farm to traditional healing, food application, and phytopharmacology. *Molecules*.

[82] Bahare S., Sharifi-Rad J., Capanoglu E. (2019). *Cucurbita* plants: from farm to industry. *Applied Sciences*.

[83] AlObaidi L. A. (2014). Study the anticancer effect of *Lepidium sativum* leaves extract on squamous cell carcinoma (CAL-27) cell lines. *Journal of Natural Science Research*.

[84] Jahani S., Heidari Z., Azami M., Moudi B. (2020). Comparison of anticancer effects of hydroalcoholic extracts of *Camellia sinensis* and *Lepidium sativum* L on hela cell line. *International Journal of Cancer Management*.

[85] Aslani E., Naghsh N., Ranjbar M. (2015). Cytotoxic effects of hydro-alcoholic extracts of cress (*Lepidium sativum*)–made from different stages of the plant-on K562 leukemia cell line. *Hormozgan Medical Journal*.

[86] Kassie F., Rabot S., Uhl M. (2002). Chemoprotective effects of garden cress (*Lepidium sativum*) and its constituents towards 2-amino-3-methyl-imidazo[4,5-f]quinoline (IQ)-induced genotoxic effects and colonic preneoplastic lesions. *Carcinogenesis*.

[87] Mahassni S. H., Al-Reemi R. M. (2013). Cytotoxic effect of an aqueous extract of *Lepidium sativum* L. seeds on human breast cancer cells. *Indian Journal of Traditional Knowledge*.

[88] Indumathy R., Aruna A. (2015). Cytotoxic potential of various extracts of *Lepidium sativum*Anticancer potential of (Linn.). An in-vitro evaluation. *International Journal of Pharmacy and Pharmaceutical Sciences*.

[89] Hussien N. A., Alsulami G. A. (2021). Anticancer potential of *Lepidium sativum* seeds aqueous extract on the azoxymethane/dextran sulfate sodium-induced colon cancer in vivo. *Current Nutraceuticals*.

[90] Ibrahim E. H., Ghramh H. A., Alshehri A. (2021). *Lepidium sativum* and its biogenic silver nanoparticles activate immune cells and induce apoptosis and cell cycle arrest in HT-29 colon cancer cells. *Journal of Biomaterials and Tissue Engineering*.

[91] Ait-Yahia O., Bouzroura S., Belkebir A., Kaci S., Aouichat A. (2015). Cytotoxic activity of flavonoid extracts from *Lepidium sativum* (Brassicaceae) seeds and leaves. *International Journal of Pharmacognosy and Phytochemical Research*.

[92] Cioboată R., Găman A., Traşcă D. (2017). Pharmacological management of non-alcoholic fatty liver disease: atorvastatin versus pentoxifylline. *Experimental and Therapeutic Medicine*.

[93] Docea A. O., Gofita E., Calina D., Ioan Z. S., Valcea D. I., Mitrut P. (2016). Autoimmune disorders due to double antiviral therapy with peginterferon and ribavirin in patients with hepatitis C virus infection. *Farmácia*.

[94] Docea A. O., Calina D., Goumenou M., Neagu M., Gofita E., Tsatsakis A. (2016). Study design for the determination of toxicity from long-term-low-dose exposure to complex mixtures of pesticides, food additives and lifestyle products. *Toxicology Letters*.

[95] Madrigal-Santillán E., Madrigal-Bujaidar E., Álvarez-González I. (2014). Review of natural products with hepatoprotective effects. *World journal of gastroenterology: WJG*.

[96] Sharifi-Rad M., Anil Kumar N. V., Zucca P. (2020). Lifestyle, oxidative stress, and antioxidants: back and forth in the pathophysiology of chronic diseases. *Frontiers in Physiology*.

[97] Wojciechowski V. V., Calina D., Tsarouhas K. (2017). A guide to acquired vitamin K coagulophathy diagnosis and treatment: the Russian perspective. *Daru*.

[98] Ahsan R., Islam K. M., Musaddik A., Haque E. (2009). Hepatoprotective activity of methanol extract of some medicinal plants against carbon tetrachloride induced hepatotoxicity in albino rats. *Global Journal of Pharmacology*.

[99] Salehi B., Sharifi-Rad J., Capanoglu E. (2019). *Cucurbita* plants: from farm to industry. *Appl. Sci.-Basel*.

[100] Sharifi-Rad J., Cruz-Martins N., López-Jornet P. (2021). Natural coumarins: exploring the pharmacological complexity and underlying molecular mechanisms. *Oxidative Medicine and Cellular Longevity*.

[101] Abuelgasim A. I., Nuha H., Mohammed A. (2008). Hepatoprotective effect of *Lepidium sativum* against carbon tetrachloride induced damage in rats. *Research Journal of Animal and Veterinary Sciences*.

[102] Rajab W. J., Ali L. H. (2020). Efficacy of *Lepidium sativum* seeds against carbon tetra chloride induced hepatotoxicity in rats. *Biochemical and Cellular Archives*.

[103] Bigoniya P., Shukla A. (2014). Phytopharmacological screening of *Lepidium sativum* seeds total alkaloid: Hepatoprotective, antidiabetic and in vitro antioxidant activity along with identification by LC/MS/MS. *PharmaNutrition*.

[104] Sakran M., Selim Y., Zidan N. (2014). A new isoflavonoid from seeds of *Lepidium sativum* L. and its protective effect on hepatotoxicity induced by paracetamol in male rats. *Molecules*.

[105] Al-Sheddi E. S., Farshori N. N., Al-Oqail M. M., Musarrat J., Al-Khedhairy A. A., Siddiqui M. A. (2016). Protective effect of *Lepidium sativum* seed extract against hydrogen peroxide-induced cytotoxicity and oxidative stress in human liver cells (HepG2). *Pharmaceutical Biology*.

[106] Yang S. C., Hsu C. Y., Chou W. L., Fang J. Y., Chuang S. Y. (2020). Bioactive agent discovery from the natural compounds for the treatment of type 2 diabetes rat model. *Molecules*.

[107] Eddouks M., Maghrani M. (2008). Effect of *Lepidium sativum* L. on renal glucose reabsorption and urinary TGF-*β*1 levels in diabetic rats. *Phytotherapy Research: An International Journal Devoted to Pharmacological and Toxicological Evaluation of Natural Product Derivatives*.

[108] Mishra N., Mohammed A., Rizvi S. (2017). Efficacy of *Lepidium Sativum* to act as an anti-diabetic agent. *Progress in Health Sciences*.

[109] Bigoniya P., Shukla A., Srivastava B. (2012). Hypoglycemic activity of *Lepidium sativum* Linn seed total alkaloid on alloxan induced diabetic rats. *Research Journal of Medicinal Plant*.

[110] Kamani M., Hosseini E. S., Kashani H. H., Atlasi M. A., Nikzad H. (2017). Protective effect of *Lepidium sativum* seed extract on histopathology and morphology of epididymis in diabetic rat model. *International Journal of Morphology*.

[111] Attia E. S., Amer A. H., Hasanein M. A. (2019). The hypoglycemic and antioxidant activities of garden cress (*Lepidium sativum* L.) seed on alloxan-induced diabetic male rats. *Natural Product Research*.

[112] Llauradó Maury G., Méndez Rodríguez D., Hendrix S. (2020). Antioxidants in plants: A valorization potential emphasizing the need for the conservation of plant biodiversity in Cuba. *Antioxidants*.

[113] Sharifi-Rad J., Rodrigues C. F., Sharopov F. (2020). Diet, lifestyle and cardiovascular diseases: linking pathophysiology to cardioprotective effects of natural bioactive compounds. *International Journal of Environmental Research and Public Health*.

[114] Bhasin P., Bansal D., Yadav O., Punia A. (2011). In vitro antioxidant activity and phytochemical analysis of seed extracts of *Lepidium sativum* a medicinal herb. *J Biosci Tech*.

[115] Golkar P., Bakhtiari M. A., Bazarganipour M. (2021). The effects of nanographene oxide on the morpho-biochemical traits and antioxidant activity of *Lepidium sativum* L. under in vitro salinity stress. *Scientia Horticulturae*.

[116] Taheri Y., Jokovic N., Vitorovic J., Grundmann O., Maroyi A., Calina D. (2021). The burden of the serious and difficult-to-treat infections and a new antibiotic available: cefiderocol. *Frontiers in Pharmacology*.

[117] Ghenea A. E., Cioboată R., Drocaş A. I. (2021). Prevalence and antimicrobial resistance of *Klebsiella* strains isolated from a county hospital in Romania. *Antibiotics*.

[118] Hussain I. (2012). Phytochemical and anti-microbial activity of *Lepidium sativum* L. *Journal of Medicinal Plant Research*.

[119] Akrayi H. F., Tawfeeq J. D. (2012). Antibacterial activity of *Lepidium sativum* and *Allium porrum* extracts and juices against some gram positive and gram negative bacteria. *Medical Journal of Islamic World Academy of Sciences*.

[120] Ibrahim N., Kebede A. (2020). *In vitro* antibacterial activities of methanol and aqueous leave extracts of selected medicinal plants against human pathogenic bacteria. *Saudi J Biol Sci*.

[121] Gacemi S., Benarous K., Imperial S., Yousfi M. (2020). Lepidine B & E as new target inhibitors from *Lepidium sativum* seeds against four enzymes of the pathogen *Candida albicans*: in vitro and in silico studies. *Endocrine, Metabolic & Immune Disorders-Drug Targets (Formerly Current Drug Targets-Immune, Endocrine & Metabolic Disorders)*.

[122] Sen S., Chakraborty R. (2017). Herbs, Gastrointestinal Protection, and Oxidative Stress. *Gastrointestinal Tissue*.

[123] Khan M. A., Khan M. A., Ahmed F. (2020). Gastrointestinal diseases segmentation and classification based on duo-deep architectures. *Pattern Recognition Letters*.

[124] Docea A. O., Mitrut P., Grigore D., Pirici D., Calina D. C., Gofita E. (2012). Immunohistochemical expression of TGF beta (TGF-*β*), TGF beta receptor 1 (TGFBR1), and Ki67 in intestinal variant of gastric adenocarcinomas. *Romanian Journal of Morphology and Embryology*.

[125] Divanji M., Lakshman K., Shylaja H., Viswanatha G., Rajesh S., Nandakumar K. (2009). Antidiarrheal activity of methanolic extracts of seeds of *Lepidium sativum*. *Journal of Natural Remedies*.

[126] Rehman N. U., Mehmood M. H., Alkharfy K. M., Gilani A. H. (2012). Studies on antidiarrheal and antispasmodic activities of *Lepidium sativum* crude extract in rats. *Phytotherapy Research*.

[127] Mehmood M. H., Alkharfy K. M., Gilani A.-H. (2011). Prokinetic and laxative activities of *Lepidium sativum* seed extract with species and tissue selective gut stimulatory actions. *Journal of Ethnopharmacology*.

[128] Gupta P. C., Pant D., Joshi P., Lohar D. R. (2010). Evaluation of antibacterial activity of *Lepidium sativum* L. seeds against food borne pathogens. *International Journal of Chemical and Analytical Science*.

[129] Adam S. I., Salih S. A., Abdelgadir W. S. (2011). " in vitro" antimicrobial assessment of" *Lepidium sativum*" L. seeds extracts. Asian. *Journal of Medical Sciences*.

[130] Omer A. B., Nour A. H., Ali M. M., Ishag O. A. O., Erwa I. Y., Ali M. A. (2020). Phytochemical screening, antimicrobial and antioxidant activity of *Lepidium sativum* seeds extract. *South Asian Research Journal of Natural Products*.

[131] Besufekad Y., Beri S., Adugnaw T., Beyene K. (2018). Antibacterial activity of Ethiopian *Lepidium sativum* L. against pathogenic bacteria. *Journal of Medicinal Plant Research*.

[132] Hala M. A., Einas H., Al-Shimaa S. (2017). Antibacterial and antioxidant properties of some selected egyptian plants. *Аннали Мечниківського інституту*.

[133] Singh V. (2017). Medicinal plants and bone healing. *National journal of maxillofacial surgery*.

[134] Salehi B., Rescigno A., Dettori T. (2020). Avocado–Soybean Unsaponifiables: A Panoply of Potentialities to Be Exploited. *Biomolecules*.

[135] bin Abdullah Juma A. B. H. (2007). The effects of *Lepidium sativum* seeds on fracture-induced healing in rabbits. *Medscape General Medicine*.

[136] Yadav Y. C., Jain A., Srivastava D., Jain A. (2011). Fracture healing activity of ethanolic extract of *Lepidium sativum* L. seeds in internally fixed rats’ femoral osteotomy model. International. *Journal of Pharmacy and Pharmaceutical Sciences*.

[137] El-Haroun H., Soliman M., El-Gawad A. (2020). Comparative study on the possible protective effect of *Lepidium Sativum* versus Teriparatide in induced osteoporosis in adult male Guinea pigs. *Egyptian Journal of Histology*.

[138] Salem E. M., Karam S. S., Kawana K. Y., Khalil N. M., Lotfy M. E. (2020). Effect of garden cress on alveolar bone healing in rats with ligature induced periodontitis (Histological& Ultrastructural study). *Alexandria Dental Journal*.

[139] Alharbi F. H., Baothman O. A. S., Zamzami M. A. (2021). Garden cress (*Lepidium sativum* L.) seeds enhancing osteogenesis postinduced-bone fracture. *Pharmacognosy Magazine*.

[140] Bakkali F., Averbeck S., Averbeck D., Idaomar M. (2008). Biological effects of essential oils - A review. *Food and Chemical Toxicology*.

[141] Batiha G. E., Alkazmi L. M., Wasef L. G., Beshbishy A. M., Nadwa E. H., Rashwan E. K. (2020). *Syzygium aromaticum* L. (Myrtaceae): traditional Uses, Bioactive Chemical Constituents, Pharmacological and Toxicological Activities. *Bioactive Chemical Constituents, Pharmacological and Toxicological Activities. Biomolecules*.

[142] Painuli S., Semwal P., Bachheti A., Bachheti R., Husen A. (2020). Nanomaterials from Non-wood Forest Products and their Applications. *Nanomaterials for Agriculture and Forestry Applications*.

[143] Salehi B., Cruz-Martins N., Butnariu M. (2021). Hesperetin's health potential: moving from preclinical to clinical evidence and bioavailability issues, to upcoming strategies to overcome current limitations. *Critical Reviews in Food Science and Nutrition*.

[144] Beshbishy A. M., Batiha G. E., Yokoyama N., Igarashi I. (2019). Ellagic acid microspheres restrict the growth of Babesia and Theileria in vitro and *Babesia microti* in vivo. *Parasites & Vectors*.

[145] El-Saber Batiha G., Magdy Beshbishy A., G Wasef L. (2020). Chemical constituents and pharmacological activities of garlic (*Allium sativum* L.): a review. *Nutrients*.

[146] Essawi T., Srour M. (2000). Screening of some Palestinian medicinal plants for antibacterial activity. *Journal of Ethnopharmacology*.

[147] Bloukh S. H., Edis Z., Sara H. A., Alhamaidah M. A. (2021). Antimicrobial properties of *Lepidium sativum* L. Facilitated Silver Nanoparticles. *Pharmaceutics.*.

[148] Yasin U., Bilal M., Bashir H., Amirzada M. I., Sumrin A., Asad M. H. H. B. (2020). Preparation and Nanoencapsulation of Lectin from *Lepidium sativum* on Chitosan- Tripolyphosphate Nanoparticle and Their Cytotoxicity against Hepatocellular Carcinoma Cells (HepG2). *BioMed Research International*.

[149] El Sayed R. A. A., Hanafy Z. E. M., Abd El Fattah H. F., Mohamed A. K. (2020). Possible antioxidant and anticancer effects of plant extracts from *Anastatica hierochuntica*, *Lepidium sativum* and *Carica papaya* against Ehrlich ascites carcinoma cells. *Cancer Biol*.

